# Concordance between *PIK3CA* mutations in endoscopic biopsy and surgically resected specimens of esophageal squamous cell carcinoma

**DOI:** 10.1186/s12885-016-3041-3

**Published:** 2017-01-09

**Authors:** Ken Hatogai, Satoshi Fujii, Takashi Kojima, Hiroyuki Daiko, Toshihiko Doi, Atsushi Ohtsu, Atsushi Ochiai, Yuichi Takiguchi, Takayuki Yoshino

**Affiliations:** 1 Department of Gastroenterology and Gastrointestinal Oncology, National Cancer Center Hospital East, 6-5-1, Kashiwanoha, Kashiwa, Chiba 277-8577 Japan; 2 Division of Pathology, Exploratory Oncology Research & Clinical Trial Center, National Cancer Center, Kashiwa, Chiba Japan; 3 Department of Medical Oncology, Graduate School of Medicine, Chiba University, Chuo-ku, Chiba Japan; 4 Department of Esophageal Surgery, National Cancer Center Hospital East, Kashiwa, Chiba Japan; 5 Exploratory Oncology Research & Clinical Trial Center, National Cancer Center, Kashiwa, Japan

**Keywords:** Esophageal squamous cell carcinoma, *PIK3CA* gene, Endoscopic biopsy, Concordance

## Abstract

**Background:**

*PIK3CA* mutations are expected to be potential therapeutic targets for esophageal squamous cell carcinoma (ESCC). We aimed to clarify the concordance between *PIK3CA* mutations detected in endoscopic biopsy specimens and corresponding surgically resected specimens.

**Methods:**

We examined five hotspot mutations in the *PIK3CA* gene (E542K, E545K, E546K, H1047R, and H1047L) in formalin-fixed and paraffin-embedded tissue sections of paired endoscopic biopsy and surgically resected specimens from 181 patients undergoing curative resection for ESCC between 2000 and 2011 using a Luminex technology-based multiplex gene mutation detection kit.

**Results:**

Mutation analyses were successfully performed for both endoscopic biopsy and surgically resected specimens in all the cases. A *PIK3CA* mutation was detected in either type of specimen in 13 cases (7.2%, 95% confidence interval: 3.9–12.0). The overall concordance rate, positive predictive value, and negative predictive value were 98.3% (178/181), 90.9% (10/11), and 98.8% (168/170), respectively. Among patients with a *PIK3CA* mutation detected in both types of specimens, the concordance between *PIK3CA* mutation genotypes was 100%. There were three cases with a discordant mutation status between the types of specimens (*PIK3CA* mutation in surgically resected specimen and wild-type in biopsy specimen in two cases, and the opposite pattern in one case), suggesting possible intratumoral heterogeneity in the *PIK3CA* mutation status.

**Conclusions:**

The *PIK3CA* mutation status was highly concordant between endoscopic biopsy and surgically resected specimens from the same patient, suggesting that endoscopic biopsy specimens can be clinically used to detect *PIK3CA* mutations in patients with ESCC.

## Background

Squamous cell carcinoma is the predominant histological subtype of esophageal cancer in Asia, whereas adenocarcinomas predominate in the United Kingdom, some other Western European countries, and the United States [1]. To date, multidisciplinary treatment approaches for esophageal cancer with different histological subtypes including surgery, chemotherapy, and radiation have been employed; however, the prognosis of these patients remains poor [2, 3]. For patients with metastatic or recurrent esophageal squamous cell carcinoma (ESCC), the available agents are quite limited. A combination of platinum agents and fluorouracil derivatives is commonly used as first-line chemotherapy, and taxanes are options for second-line chemotherapy [1, 4]. In addition, no molecular-target therapies have been established for the treatment of ESCC. Therefore, there is an unmet medical need for ESCC treatment, particularly for patients who are in good physical condition but who are refractory or intolerant to standard therapies.

The phosphoinositide 3-kinase (PI3K)–Akt–mammalian target of rapamycin (mTOR) pathway plays a pivotal role in cancer cell proliferation, and mutations in the *PIK3CA* gene are commonly found in various cancers regardless of histological subtypes [5]. More than 80% of *PIK3CA* mutations occur in two major regions: the helical domain (exon 9), and the kinase domain (exon 20); moreover, three mutations (E542K, E545K, and H1047R) have been regarded as hotspot mutations [6]. In a phase 1 trial evaluating an mTOR inhibitor, a case with advanced ESCC exhibited a partial response, although the *PIK3CA* mutation status was unknown [7]. In addition, *PIK3CA* mutations have been suggested to be a potential predictive biomarker for PI3K–Akt–mTOR inhibitors in a review of early phase clinical trials for the testing of such agents in various solid cancers [8]. In this report, a case with squamous cell head and neck carcinoma, which is genetically similar to ESCC, harboring a *PIK3CA* mutation (H1047R) demonstrated a partial response to a PI3K–Akt–mTOR inhibitor. The frequency of *PIK3CA* mutations in ESCC has been reported to range from 2.2 to 21% [9–16], whereas mutations in genes in the RAS–RAF pathway are very rare [15, 17, 18]. Accordingly, *PIK3CA* mutations may be a potential target molecule in ESCC treatment.

Previous studies investigating the frequency of *PIK3CA* mutations in ESCC used available clinical samples obtained from either surgically resected specimens or biopsy specimens [9–16]. Clarifying whether *PIK3CA* mutations from biopsy specimens can be detected in corresponding surgically resected specimens is important for the future clinical development of ESCC treatment. Therefore, the present study examined the frequency of *PIK3CA* mutations and the concordance between *PIK3CA* mutations detected in endoscopic biopsy specimens and those detected in corresponding surgically resected specimens in patients with ESCC.

## Methods

### Patients

Among 352 previously untreated patients with ESCC who underwent a curative-intent transthoracic esophagectomy with extended lymphadenectomy at the National Cancer Center Hospital East, Kashiwa, Japan, between January 2000 and December 2011, a total of 181 patients were enrolled according to the following selection criteria: (i) pathological T factor of at least T1b, (ii) availability of paired samples of endoscopic biopsy and surgically resected specimens, (iii) patient age of ≤75 years, (iv) absence of past or concurrent history of cancer, (v) adequate organ function, and (vi) absence of in-hospital death following surgery.

### Tissue samples

**Fig. 1:**
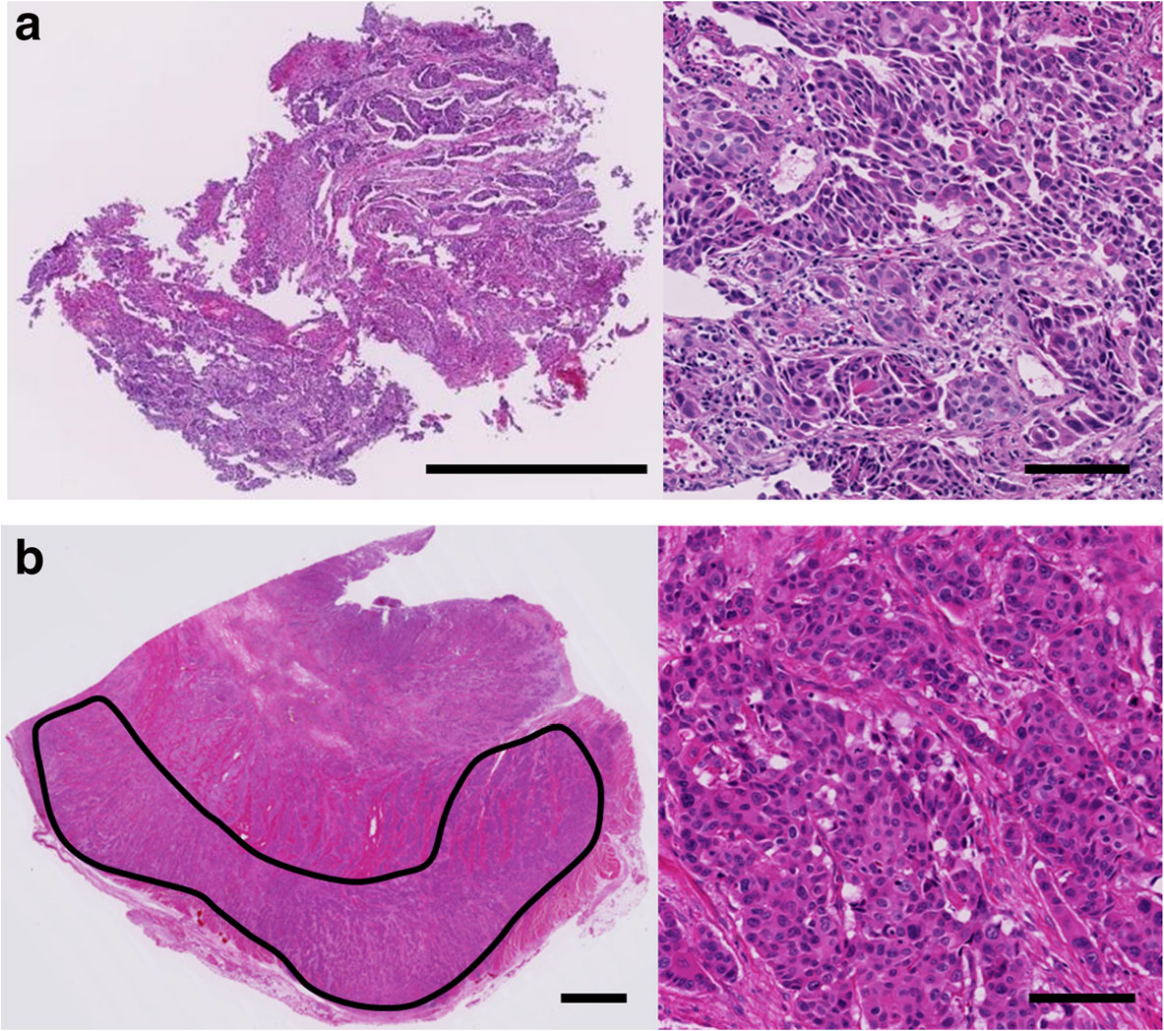
Photomicrographs of a case harboring a *PIK3CA* mutation in both types of specimens (H1047R; Case No. 66). Paired endoscopic biopsy and surgically resected specimens show the same mutation. **a** The entire biopsy was manually microdissected (scale bar: 1 mm). **b** The tumor area in the surgically resected specimen was marked (indicated by the *solid line*) and manually microdissected (scale bar: 2 mm). High-power views of each specimen are shown in the *right panels* (scale bar: 100 μm)

Archival formalin-fixed and paraffin-embedded (FFPE) tissue sections of paired endoscopic biopsy and surgically resected specimens from the enrolled patients were used for DNA extraction. Thin tissue sections (4 μm) cut from an FFPE tissue block were placed on microscopic slides and stained with hematoxylin and eosin (H&E) for histological examination. Five unstained tissue sections (10 μm) were also continuously cut from the same tissue block and were placed on a glass slide. The tumor histology was confirmed by a pathologist specializing in gastrointestinal cancer (SF) based on a microscopic examination of the H&E-stained slides. For the endoscopic biopsy specimens, the entire biopsy specimen was manually microdissected after confirming that the ratio of tumor cells to whole cells was >20% on slides stained with H&E. For the surgically resected specimens, a tumor area with a small amount of stromal cells where the ratio of tumor cells to whole cells was >50% on slides stained with H&E was manually microdissected (Fig. 1). DNA extraction was performed using the heat-induced retrieval method, as described previously [19, 20].

### *PIK3CA* mutation analysis

*PIK3CA* mutations were detected using a Luminex (xMAP) technology-based multiplex gene mutation detection kit (GENOSEARCH Mu-PACK; MBL, Nagoya, Japan), which was developed prior to the present study; an optimal concordance between the kit and the conventional direct sequencing method was confirmed previously [21]. A total of five *PIK3CA* mutations, including codon 542 (E542K), codon 545 (E545K), and codon 546 (E546K) in exon 9 and codon 1047 (H1047R, H1047L) in exon 20, were investigated. The lowest detection limit of the percentage of mutant allele was 5%. The protocol details have been described previously [21]. Next-generation sequencing (NGS, Ion Ampliseq™ Cancer Hotspot Panel v2; Life Technologies, Carlsbad, CA, USA) was additionally performed using DNA obtained from the same FFPE block for cases in which the mutation status of the endoscopic biopsy specimen and the surgically resected specimen differed when assessed using the method described above. In cases where NGS was not successfully performed, DNAs were extracted from two separate tumor portions of the same FFPE block as that used in the primary analysis, and *PIK3CA* mutations in each portion were measured using the kit.

### Statistical analysis

The *PIK3CA* mutation frequency was determined as the proportion of specimens with a *PIK3CA* mutation among either all the endoscopic biopsy or all the surgically resected specimens. In addition to the mutation frequency, the overall concordance rate, positive concordance rate, negative concordance rate, positive predictive value, and negative predictive value of the endoscopic biopsy specimens, compared with the surgically resected specimens as a reference, were evaluated and presented with the 95% confidence intervals (CI) [22]. The associations between the *PIK3CA* mutation status and clinicopathological factors were assessed using the *t*-test for continuous variables and the chi-square test for categorical variables or the Fisher’s exact test for dichotomous variables. All *P* values were reported as two-sided, with a significance level of 0.05. All statistical analyses were performed using IBM SPSS statistics 20 (IBM Japan Ltd., Tokyo, Japan).

## Results

### Patients

The clinicopathological characteristics of the enrolled patients are shown in Table 1. The mean age of the patients was 62.7 years, with the majority of the patients being male (82.9%). Most of the patients had T2 or T3 disease, but patients with T1b disease (28.2%) and curatively resected T4 disease (2.2%) were included. One hundred twenty-five patients (68.5%) had lymph node metastases, while 56 (31.5%) did not. Eleven patients (6.1%) had non-regional lymph node metastases (M1) but not distant organ metastases and were judged as Stage IV. In total, 362 samples from 181 patients were included in the concordance analysis.

**Table 1:** Clinicopathological characteristics of the patients

Characteristics	Number	Percent
Age (mean ± SD)	62.7 ± 7.0	
Gender		
Male	150	82.9
Female	31	17.1
Smoking habit		
Non-smoker	33	18.2
Smoker	148	81.8
Alcohol consumption		
Non-drinker	33	18.2
Drinker	148	81.8
Location		
Upper	26	14.4
Middle	83	45.9
Lower	72	39.8
T factor		
1b	51	28.2
2	22	12.2
3	104	57.5
4	4	2.2
N factor		
0	57	31.5
1	60	33.1
2	48	26.5
3	16	8.8
M factor		
0	170	93.9
1	11	6.1
TNM stage		
I	30	16.6
II	51	28.2
III	89	49.2
IV	11	6.1
Histological grade		
W/D	38	21.0
M/D	120	66.3
P/D	23	12.7
Lymphatic invasion		
Absent	89	49.2
Present	92	50.8
Venous invasion		
Absent	36	19.9
Present	145	80.1

*Abbreviations*: *W/D* well differentiated, *M/D* moderately differentiated, *P/D* poorly differentiated, *SD* standard deviation

### Frequency of *PIK3CA* mutations

The median amount of extracted DNA was 11,268 ng (interquartile range [IQR]: 6558–18,268) for the surgically resected specimens and 2452 ng (IQR: 1748–3352) for the biopsy specimens. For quality control, we checked the absorbance at 260 nm (A_260_) and 280 nm (A_280_), and the median A_260_/ A_280_ scores were 1.91 (IQR: 1.85–1.98) and 1.93 (IQR: 1.88–1.97) for the endoscopic biopsy and surgically resected specimens, respectively. Mutation analyses were successfully performed for both the endoscopic biopsy and surgically resected specimens in all the cases. Among the endoscopic biopsy specimens, there were 11 cases with *PIK3CA* mutations: two with E542K, three with E545K, five with H1047R, and one with H1047L. Among the surgically resected specimens, there were 12 cases with *PIK3CA* mutations: one with E542K, four with E545K, six with H1047R, and one with H1047L. Overall, the *PIK3CA* mutation frequency based on a positive mutation status for either specimen was 7.2% (13/181, 95% CI: 3.9–12.0) (Table 2). Figure 1 shows micrographs of the ESCC case that harbored a *PIK3CA* mutation (H1047R) in both the biopsy and the surgically resected specimens. DNA was isolated from the entire biopsy specimen and the area marked by the solid line in the surgically resected specimen. No histological findings specific to cases with a *PIK3CA* mutation were observed in the present study. There were three cases with discordant results between the endoscopic biopsy and the surgically resected specimens.

**Table 2:** Cases with *PIK3CA* mutations in either endoscopic biopsy or surgically resected specimens

Case	Endoscopic biopsy		Surgical resection		T factor
	Mutation	Histology	Mutation	Histology	
8	E542K	M/D	Wild type	M/D	T3
50	E545K	M/D	E545K	M/D	T1b
61	Wild type	P/D	E545K	M/D	T4
66	H1047R	M/D	H1047R	P/D	T3
75	H1047R	M/D	H1047R	M/D	T3
87	E542K	M/D	E542K	M/D	T3
111	E545K	M/D	E545K	M/D	T3
114	E545K	M/D	E545K	M/D	T3
127	H1047R	M/D	H1047R	M/D	T3
132	Wild type	M/D	H1047R	M/D	T1b
140	H1047L	M/D	H1047L	M/D	T3
163	H1047R	M/D	H1047R	M/D	T3
166	H1047R	M/D	H1047R	W/D	T1b

*Abbreviations*: *W/D* well differentiated, *M/D* moderately differentiated, *P/D* poorly differentiated

The correlations between clinicopathological factors and the *PIK3CA* mutation status as detected using DNA extracted from either the endoscopic biopsy or surgically resected specimens are presented in Table 3. No clear differences were observed between the *PIK3CA* mutation status and the clinicopathological factors that were examined.

**Table 3:** Relationship between clinicopathological characteristics and *PIK3CA* mutation status

Characteristics	*PIK3CA* mutation status				*P* value
	Wild-type		Mutant		
	Number	%	Number	%	
Mean age ± SD	62.8 ± 7.1		61.9 ± 5.6		0.643
Gender					0.469
Male	140	83.3	10	76.9	
Female	28	16.7	3	23.1	
Smoking status					0.468
Non-smoker	32	19.0	1	7.7	
Smoker	136	81.0	12	92.3	
Alcohol consumption					1.000
Non-drinker	14	8.3	1	7.7	
Drinker	154	91.7	12	92.3	
Location					1.000
Upper/Middle	101	60.1	8	61.5	
Lower	67	39.9	5	38.5	
T factor					0.247
1–2	70	41.7	3	23.1	
3–4	98	58.3	10	76.9	
Lymph node metastases					1.000
Absent	52	31.0	4	30.8	
Present	116	69.0	9	69.2	
TNM stage					0.775
I-II	76	45.2	5	38.5	
III-IV	92	54.8	8	61.5	
Histological differentiation					1.000
W/D, M/D	146	86.9	12	92.3	
P/D	22	13.1	1	7.7	
Lymphatic invasion					0.400
Absent	81	48.2	8	61.5	
Present	87	51.8	5	38.5	
Venous invasion					0.470
Absent	35	20.8	1	7.7	
Present	133	79.2	12	92.3	

*Abbreviations*: *SD* standard deviation, *W/D* well differentiated, *M/D* moderately differentiated, *P/D* poorly differentiated

### Concordance analysis

As shown in Table 4, 168 cases and 10 cases were determined to have a wild-type and a mutant-type, respectively, in both the endoscopic biopsy and surgically resected specimens. In contrast, a concordant result was not achieved in the remaining three cases. The overall concordance rate for the *PIK3CA* mutation status between the endoscopic biopsy and surgically resected specimens was 98.3% ([168 + 10]/181, 95% CI: 95.2–99.7). The positive and negative concordance rates were 83.3% (10/12, 95% CI: 51.6–97.9) and 99.4% (168/169, 95% CI: 96.7–99.9), respectively. The positive and negative predictive values were 90.9% (10/11, 95% CI: 58.7–99.8) and 98.8% (168/170, 95% CI: 95.8–99.9), respectively.

**Table 4:** Concordance of *PIK3CA* mutation status between endoscopic biopsy and surgically resected specimens

		Surgically resected specimens (reference method)		
		Wild type	Mutant type	Total
Endoscopic biopsy specimens (trial method)	Wild type	168	2	170
	Mutant type	1	10	11
	Total	169	12	181
Overall concordance rate		98.3% (95% CI: 95.2–99.7)		
Positive concordance rate		83.3% (95% CI: 51.6–97.9)		
Negative concordance rate		99.4% (95% CI: 96.7–99.9)		
Positive predictive value		90.9% (95% CI: 58.7–99.8)		
Negative predictive value		98.8% (95% CI: 95.8–99.9)		

*Abbreviations*: *CI* confidence interval

The *PIK3CA* mutation genotypes in the endoscopic biopsy and surgically resected specimens obtained for the ten cases that exhibited a *PIK3CA* mutation in both specimen types are compared in Table 5. The concordance of the *PIK3CA* mutation genotype was 100% (10/10, 95% CI: 74%–100%).

**Table 5:** Concordance of *PIK3CA* mutation genotypes between endoscopic biopsy and surgically resected specimens in cases exhibiting a *PIK3CA* mutation in both specimen types

		Surgically resected specimens (reference method)				
		E542K	E545K	E546Q	H1047R	H1047L
Endoscopic biopsy specimens (trial method)	E542K	1	0	0	0	0
	E545K	0	3	0	0	0
	E546Q	0	0	0	0	0
	H1047R	0	0	0	5	0
	H1047L	0	0	0	0	1

### Details of discrepancy of *PIK3CA* mutation status between endoscopic biopsy and surgically resected specimens

**Fig. 2:**
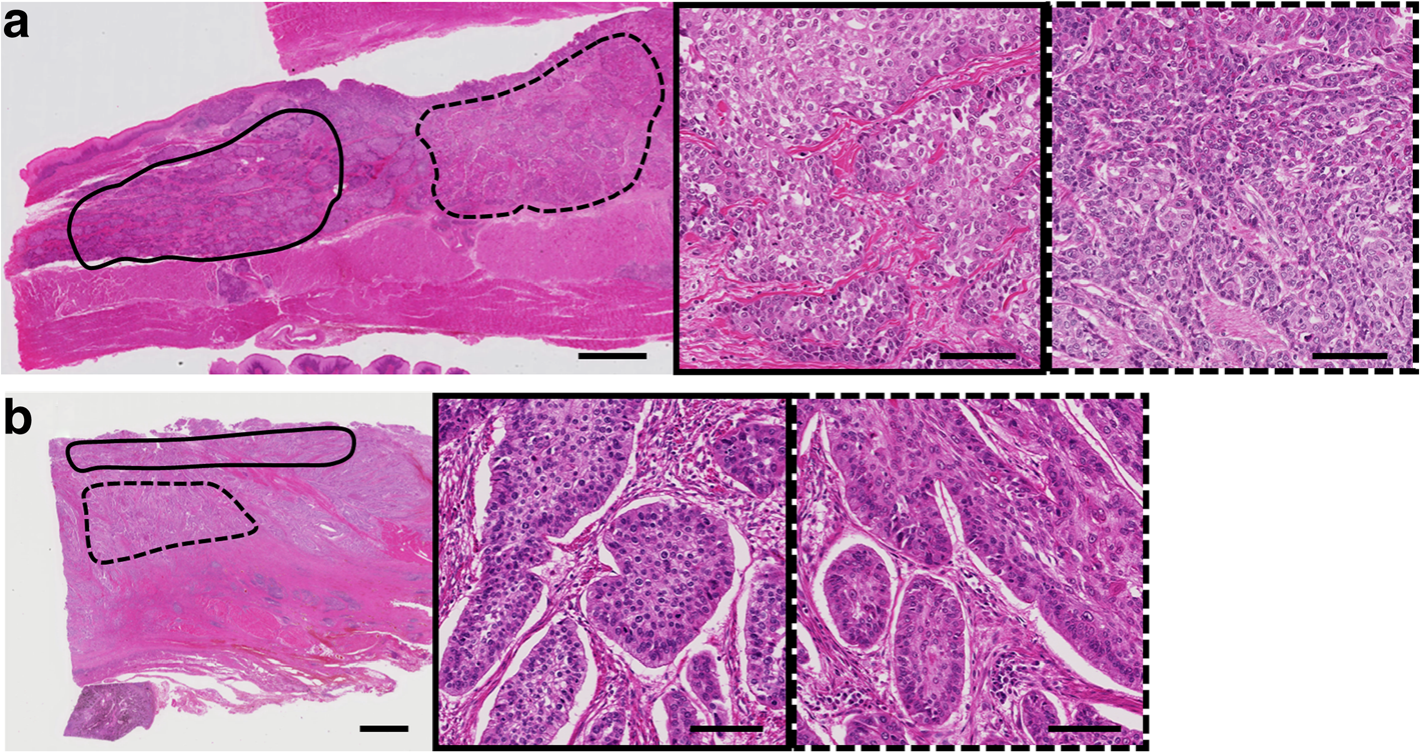
Photomicrographs of two cases with a heterogeneous *PIK3CA* mutation status. A *PIK3CA* mutation was detected in the portions marked by the *dotted lines*, but not in the portion marked by the *solid lines* (scale bar: 2 mm). **a** Case No. 8 and **b** Case No. 61. High-power views of each portion indicated by the *solid* and *dotted lines* are shown in the insets (scale bar: 100 μm)

The details of the three cases with a discordant *PIK3CA* mutation status were as follows: two cases had mutation-positive surgical specimens but exhibited a wild-type profile for their biopsy specimens (Case No. 61 and No. 132), while the opposite pattern was observed in one case (Case No. 8; Table 2). We performed NGS for these three cases; however, the DNA amplification was incomplete and the sequencing was not successful in both Case No. 8 and Case No. 61. In Case No. 132, NGS revealed a *PIK3CA* mutation in H1047R with a mutant allele frequency of 4.5% in the surgically resected specimen; no *PIK3CA* mutations were detected in the endoscopic biopsy specimen. In Case No. 8 and Case No. 61, *PIK3CA* mutations were detected in only one portion and were not detected in the other when two separate tumor portions from the same FFPE block were analyzed (Fig. 2).

## Discussion

To the best of our knowledge, this is the first report to investigate the concordance of the *PIK3CA* mutation status between endoscopic biopsy and surgically resected specimens using FFPE tissues from patients with ESCC. In the present study, we demonstrated that, although not frequent, a certain proportion of patients with ESCC harbored *PIK3CA* mutations, and the mutation statuses of the two types of specimens were highly concordant.

Among the five hotspot mutations assessed in the present study, E542K, E545K and E546K are located in exon 9, corresponding to the helical domain, and H1047R and H1047L are located in exon 20, corresponding to the kinase domain. The mutations in both of these domains increase the kinase activity of PI3K and activate the PI3K–Akt–mTOR pathway, resulting in the activation of cell signaling and the promotion of cell growth and invasion [23, 24]. The present study showed no significant association between the *PIK3CA* mutation status and the clinicopathological characteristics of the ESCC cases, suggesting that an examination of the clinicopathological factors prior to genetic analysis might not be capable of predicting the presence of a *PIK3CA* mutation. In other words, ESCC patients with a *PIK3CA* mutation constitute a subgroup only in terms of the applicability of a PI3K inhibitor, since emerging evidence suggests that patients with *PIK3CA*-mutated cancer might benefit from treatment with PI3K inhibitors [8, 25].

The high overall concordance rate (98.3%) between endoscopic biopsy and surgically resected specimens observed in this study suggests that *PIK3CA* mutations are homogeneously distributed in the primary tumor in most cases. Although the intratumoral heterogeneity of the *PIK3CA* mutation status has not yet been investigated for ESCC, the results of our study are in line with the homogeneous distribution of the *PIK3CA* mutation status in the primary tumor observed in colorectal and breast cancers [26, 27]. In colorectal cancer, the concordance rate of the *KRAS* mutation status between endoscopic biopsy and surgically resected specimens is high [28], similar to that of the *PIK3CA* mutation status in ESCC demonstrated in the present study, and endoscopic biopsy specimens are used for the molecular analysis of *KRAS* mutations to evaluate the clinical indications for anti-epidermal growth factor receptor antibody therapy. These findings suggest that FFPE clinical samples obtained from endoscopic biopsies are applicable to the identification of *PIK3CA* mutations in ESCC. In contrast, the discordant mutation status between endoscopic biopsy and surgically resected specimens that was observed in three cases may be attributable to intratumoral heterogeneity. Because endoscopic biopsy specimens represent a limited and superficial sampling of the primary tumor, intratumoral heterogeneity can be an obstacle to establishing a precise biomarker diagnosis. Obtaining multiple endoscopic biopsy samples from primary tumors may improve the likelihood of detecting a mutation and may minimize potential mutational discordances.

The *PIK3CA* mutation frequency of 7.2% observed in this study is based on Luminex (xMAP) technology targeting five hotspot mutations in the *PIK3CA* gene with a detection limit of 5% and is compatible with the COSMIC database published by the Sanger institute (9.5%), and with those of the previous studies ranging from 2.2 to 21% [9–16]. The variation in mutation frequency among these studies is thought to be mainly attributable to differences in the methods used to detect *PIK3CA* mutations as well as differences in the patient cohorts, with factors such as disease stage, prior therapy, and ethnicity playing major roles. The frequency of *PIK3CA* mutations has been reported to be 2.2%–7.7% using direct sequencing, 11.5%–21% using pyrosequencing or other high-sensitivity methods, and 4.5%–9.0% using NGS [9–12, 14–16, 29, 30]. The results of the present study were likely influenced by the sensitivity of the mutation testing. Collecting a sufficient number of cancer cells and excluding non-cancerous cells from biopsy specimens is difficult using manual microdissection for DNA extraction because the original size of the biopsy specimens is considerably smaller than that of the surgically resected specimens. One strategy is to use a detection method with a higher sensitivity, thereby reducing the risk of missing a relatively small fraction of cancer cells carrying a *PIK3CA* mutation. However, the relationship between the proportion of cancer cells with a *PIK3CA* mutation and biological differences has not yet been reported, and the relationship between the proportion of cancer cells with a *PIK3CA* mutation and differences in the response to PI3K inhibitors for any cancer type, including ESCC, remains unknown. Importantly, the present study revealed a case in which a *PIK3CA* mutation was observed in a biopsy specimen, but the wild-type was observed in the surgically resected specimen. This event suggests the existence of another problem in the detection of *PIK3CA* mutations: that is, heterogeneity. In addition to the sensitivity of mutation testing, tumor heterogeneity is also likely to influence the detection sensitivity. Since the efficacy of agents inhibiting the PI3K–Akt–mTOR pathway has been demonstrated clinically, further investigation of the optimal detection method and its detection limit is needed to ensure that patients who might benefit from such treatment are accurately identified.

Recently, phase 1, phase 2, and phase 3 clinical trials examining a number of PI3K inhibitors have begun for patients with various types of cancer [31, 32]. Among these agents, the clinical efficacy of buparlisib (BKM120), a pan-PI3K inhibitor, has been demonstrated in breast cancer patients in a phase 3 trial, and the presence of a *PIK3CA* mutation was shown to predict a response to this agent [25]. Several phase 2 trials examining buparlisib in ESCC patients are also currently ongoing (registration ID: NCT01806649, UMIN000011217). Additional biomarker studies performed during these clinical trials may reveal whether the *PIK3CA* mutation can be used as a biomarker to predict the efficacy of PI3K inhibitors in patients with ESCC.

Although heterogeneity in the *PIK3CA* mutation status between primary tumors and corresponding lymph nodes or distant organ metastases is reportedly rare for colorectal and breast cancers, limited information is available with regard to ESCC. For the development of therapies targeting the *PIK3CA* mutation in patients with ESCC, the potential for heterogeneity between primary tumors and metastases must be further investigated.

## Conclusion

The detection of *PIK3CA* mutations could be used to define a subset of patients who may be potential candidates for treatment with inhibitors of the PI3K–Akt–mTOR pathway. The *PIK3CA* mutation status was highly concordant between endoscopic biopsy and surgically resected specimens in patients with ESCC, suggesting that endoscopic biopsy specimens are clinically applicable for the detection of *PIK3CA* mutations.

## Abbreviations

CI: Confidence intervals
ESCC: Esophageal squamous cell carcinoma
FFPE: Formalin-fixed and paraffin-embedded
H&E: Hematoxylin–eosin
IQR: Interquartile range
mTOR: Mammalian target of rapamycin
PI3K: Phosphoinositide 3-kinase
